# Galectin-3 as a Therapeutic Target for NSAID-Induced Intestinal Ulcers

**DOI:** 10.3389/fimmu.2020.550366

**Published:** 2020-09-23

**Authors:** Ah-Mee Park, Sundar Khadka, Fumitaka Sato, Seiichi Omura, Mitsugu Fujita, Daniel K. Hsu, Fu-Tong Liu, Ikuo Tsunoda

**Affiliations:** ^1^Department of Microbiology, Faculty of Medicine, Kindai University, Osaka, Japan; ^2^Department of Dermatology, University of California Davis Health System, Sacramento, CA, United States; ^3^Institute of Biomedical Sciences, Academia Sinica, Taipei, Taiwan

**Keywords:** adverse effect, animal model, cyclooxygenase-2 inhibitors, microbiome, PAS stain, small intestine, 16S rRNA, gastrointestinal flora

## Abstract

Non-steroidal anti-inflammatory drugs (NSAIDs) induce ulcers in the gastrointestinal tract, including the stomach and small intestine. NSAID-induced gastric ulcers can be prevented by taking acid-neutralizing/inhibitory drugs and cytoprotective agents. In contrast, there are no medicines to control NSAID-induced small intestinal ulcers, which are accompanied by a mucosal invasion of bacteria and subsequent activation of immune cells. Galectin-3 (Gal3), an endogenous lectin, has anti-microbial and pro-inflammatory functions. In the small intestine, since Gal3 is highly expressed in epithelial cells constitutively and macrophages inducibly, the Gal3 level can affect microbiota composition and macrophage activation. We hypothesized that the modulation of Gal3 expression could be beneficial in NSAID-induced intestinal ulcers. Using Gal3 knockout (Gal3KO) mice, we determined whether Gal3 could be a therapeutic target in NSAID-induced intestinal ulcers. Following the administration of indomethacin, an NSAID, we found that small intestinal ulcers were less severe in Gal3KO mice than in wild-type (WT) mice. We also found that the composition of intestinal microbiota was different between WT and Gal3KO mice and that bactericidal antibiotic polymyxin B treatment significantly suppressed NSAID-induced ulcers. Furthermore, clodronate, a macrophage modulator, attenuated NSAID-induced ulcers. Therefore, Gal3 could be an exacerbating factor in NSAID-induced intestinal ulcers by affecting the intestinal microbiota population and macrophage activity. Inhibition of Gal3 may be a therapeutic strategy in NSAID-induced intestinal ulcers.

**Clinical Trial Registration:**

www.ClinicalTrials.gov, identifier NCT03832946.

## Introduction

Although non-steroidal anti-inflammatory drug (NSAID) therapy has been used to suppress inflammation and pain, it sometimes induces potentially life-threatening complications related to NSAID-induced gastrointestinal ulcers (1–3). In the stomach, although NSAID-induced ulcers are occasionally fatal, it can be prevented/treated by taking acid-neutralizing/inhibitory drugs and cytoprotective agents. In the small intestine, NSAIDs can also induce ulcers, resulting in bleeding and perforation (1, 3). Unlike the stomach ulcers, there is no treatment to control NSAID-induced small intestinal ulcers.

Galectin-3 (Gal3) is one of the galectin family members, which is highly expressed by activated macrophages as well as various cell types constitutively including gastrointestinal epithelial cells (4, 5). Gal3 has been shown to have a variety of pro-inflammatory and anti-microbial functions (6–11). For example, activated macrophages have been shown to express Gal3, which plays roles in not only the survival and phagocytosis of macrophages/neutrophils (6–8), but also neutrophil extravasation (9–11). Gal3 can be directly bacteriostatic for *Helicobacter pylori* (12), *Staphylococcus pneumonia* (9), and cytocidal for *Candida albicans* (13). On the other hand, Gal3 is known to be an exacerbating factor in several diseases experimentally and clinically, including idiopathic pulmonary fibrosis (14, 15), non-alcoholic steatohepatitis with cirrhosis (16), and ovarian carcinoma (17). Thus, Gal3 is considered as a therapeutic target for these diseases (18), in which the development of Gal3 inhibitors has been attempted.

Non-steroidal anti-inflammatory drugs-induced small intestinal ulcers have been proposed to develop with several factors: a decrease in mucus secretion caused by low prostaglandin synthesis; the mucosal invasion of bacteria; and activation of immune cells including macrophages (19, 20). Since Gal3 has pro-inflammatory and anti-microbial functions, changes in the Gal3 levels can affect immune cell activation and bacterial composition in the intestine. Here, we hypothesize that the modulation of Gal3 expression can be beneficial in NSAID-induced intestinal ulcers. In the following sections, we will introduce our experimental findings, in which small intestinal ulcers were suppressed in Gal3 knockout (Gal3KO) mice following administration of indomethacin (Indo), an NSAID. We will propose that the inhibition of Gal3 can be a therapeutic strategy in NSAID-induced intestinal ulcers.

## Galectin-3 in Intestinal Ulcers

### Attenuation of NSAID-Induced Small Intestinal Ulcers in Gal3KO Mice

**FIGURE 1:**
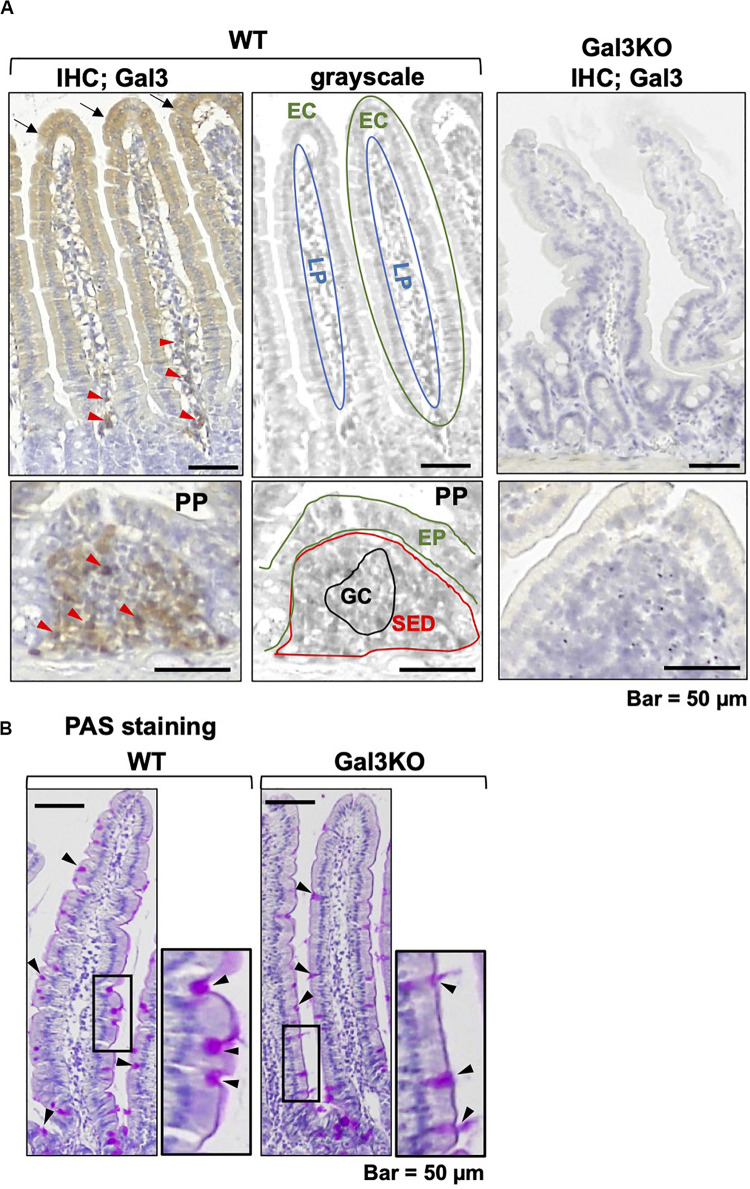
Galectin-3 (Gal3) and mucin staining. **(A)** We conducted immunohistochemistry with anti-Gal3 antibody (BioLegend, San Diego, CA, United States) using a Histofine SAB-PO kit (Nichirei Biosciences; Tokyo, Japan), in 4-μm thick small intestine sections of wild-type (WT) and Gal3 knockout (Gal3KO) mice. Gal3 was stained brown, and nuclei were counterstained with hematoxylin (blue). In WT mice, we found moderate Gal3 staining in the cytoplasm of enterocytes (EC, black arrows) and intense staining of mononuclear cells (red arrowheads) in the lamina propria (LP). In the Peyer’s patch (PP), Gal3 positive cells were detected in the subepithelial dome region (SED), but not in the germinal center (GC). Gal3KO mice had no Gal3 positive cells. The middle grayscale panels were shown to indicate anatomical structures. EP, epithelium. **(B)** Periodic acid-Schiff (PAS) staining of intestine sections of WT and Gal3KO mice. The cytoplasm of goblet cells (black arrowheads) and the luminal surface of enterocytes were stained purple-magenta due to the presence of mucins.

We first examined Gal3 expression in the small intestine (Figure 1A) in 10–14 week-old wild-type (WT) CD1 mice (Charles River Laboratories Japan, Yokohama, Japan) and Gal3KO CD1 mice (12). In WT mice, enterocytes of the small intestine moderately expressed Gal3 in the cytoplasm. On the other hand, mononuclear cells in the lamina propria (LP) and subepithelial dome region (SED) of the Peyer’s patch (PP) highly expressed Gal3. We confirmed that Gal3KO mice had no Gal3 expression. Although Gal3 has been reported to play a role in protein trafficking and morphogenesis of enterocytes of the small intestine (21), we found no obvious morphological changes in the small intestine of Gal3KO mice. We also assessed the intestinal mucus level with periodic acid-Schiff (PAS) stain, by which mucus is stained purple-magenta (Figure 1B). PAS-positive mucus was observed in the cytoplasm of goblet cells and the luminal surface of the enterocytes. We found similar numbers of goblet cells and thickness of PAS-positive mucus in WT and Gal3KO mice.

**FIGURE 2:**
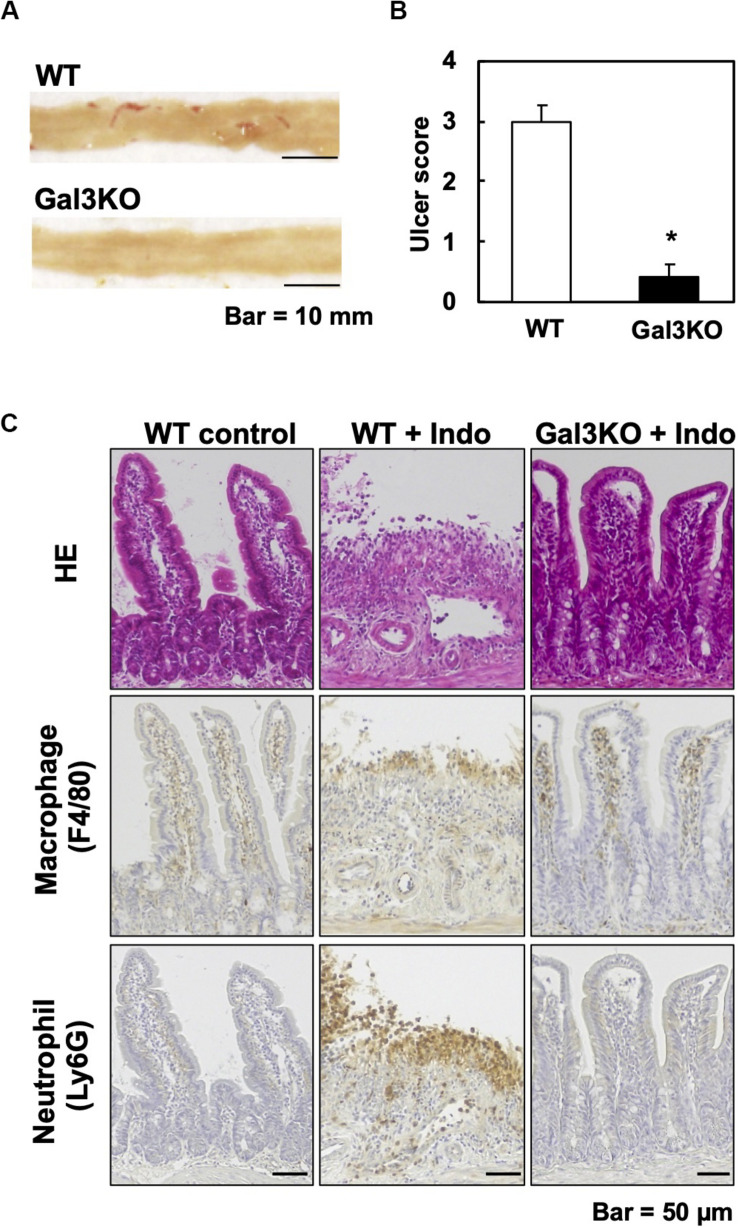
Indomethacin (Indo)-induced small intestinal ulcers. We treated WT and Gal3KO mice with Indo, and 18 h later, harvested the small intestines (*n* = 7 per group). We determined the Indo concentration 5 mg/kg body weight by “Human equivalent dose calculation” (42). Indo (FUJIFILM Wako, Osaka, Japan) was solved in a 0.5% NaHCO_3_ solution and administrated to non-fasted mice by using a stomach tube. **(A)** We opened the small intestine along the anti-mesenteric side, and took macroscopic images with a scale by a digital camera (Canon, Tokyo, Japan). Representative macroscopic images of the jejunum from WT (ulcer score = 3) and Gal3KO mice (ulcer score = 0). Bar = 10 mm. **(B)** The ulcer severity was assessed using the ulcer score (23) with modification. The macroscopic ulceration areas were captured and quantified by an ImageJ software (NIH, Bethesda, MD, United States) and summed per jejunum. The entire jejunum areas were similar in all mice examined and were around 1,200 mm^2^. We used the modified ulcer score as follows: 0 = no lesion, 1 = less than 5 mm^2^, 2 = 5∼20 mm^2^, 3 = 21∼40 mm^2^, 4 = 41∼70 mm^2^, and 5 = more than 71 mm^2^. *P < 0.05 by the Mann–Whitney *U* test. **(C)** Small intestine lesions, 18 h after Indo administration. We stained formalin-fixed paraffin sections with hematoxylin and eosin, anti-F4/80 antibody (AbD Serotec, Kidlington, United Kingdom) for macrophages, and anti-Ly6G antibody (BD Biosciences, San Jose, CA, United States) for neutrophils. Bar = 50 μm.

**FIGURE 3:**
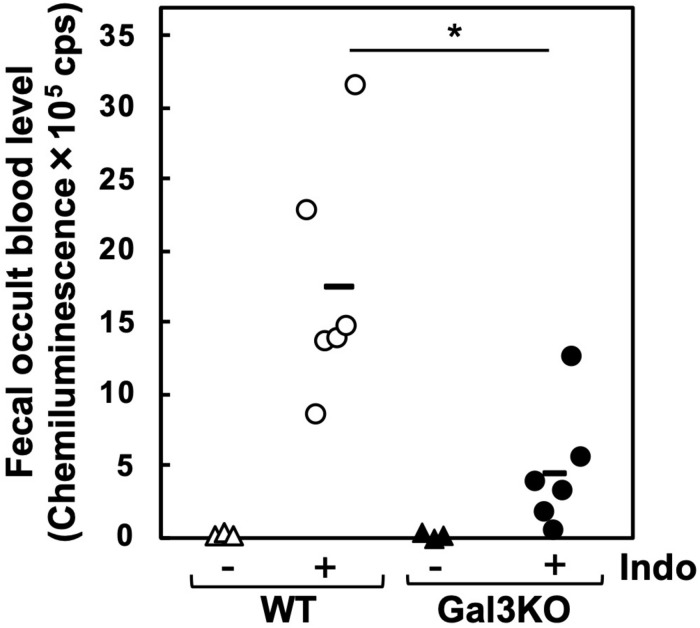
Fecal occult blood (FOB) of Indo-administered mice. FOB levels were higher in Indo-administered WT mice (○) than in Gal3KO mice (●). Without Indo administration, FOB was not detectable in WT mice (△) or Gal3KO mice (▲). +Indo groups, *n* = 7; –Ind groups, *n* = 3. *P < 0.05 WT+Indo versus Gal3KO+Indo by the Student *t-*test; count per second. FOB level was determined as described previously (43). Feces were suspended in distilled water and centrifuged at 12,000 × *g*. The supernatant was mixed with the luminol reagent (FUJIFILM Wako), and then chemiluminescence was measured by a luminometer (Wallac ARVO SX 1420 multilabel counter, PerkinElmer, Waltham, MA, United States).

Experimentally, a mouse model for small intestinal ulcers has been induced with oral administration of Indo to conventionally fed mice without fasting; this regimen does not induce ulcers in the stomach (22). To examine the roles of Gal3 in the small intestine, we administrated Indo to WT and Gal3KO mice, harvested the gastrointestinal tissues, and identified ulcers macroscopically. We detected ulcers predominantly in the jejunum, but not in the ileum; there was no evident ulcer in the stomach or colon of these mice, which was most likely due to the low dosage of Indo administered to conventionally fed (no-fasted) mice (22). We quantified the severity of ulcers in the jejunum using the ulcer score (23), and found that Gal3KO mice had significantly lower ulcer scores than WT mice (Figures 2A,B). Microscopically, we found severe ulceration in the WT mice treated with Indo (WT+Indo) (Figure 2C). Although F4/80^+^ macrophages were detected in the lamina propria in all mice, F4/80^+^ macrophages were increased only in the ulcerated lesions of the WT+Indo. Ly6G^+^ neutrophils were accumulated in the ulcerated lesions of the WT+Indo but not detectable in the control WT mice or the Gal3KO mice treated with Indo (Gal3KO+Indo). We also assessed the severity of ulcers by the fecal occult blood (FOB) levels. We found that the FOB levels were significantly lower in the Gal3KO+Indo mice than in the WT+Indo mice, which were associated with the ulcer scores (Figure 3). Without Indo administration, FOB was not detected.

### Role of Microbiota in NSAID-Induced Ulcers in WT and Gal3KO Mice

**FIGURE 4:**
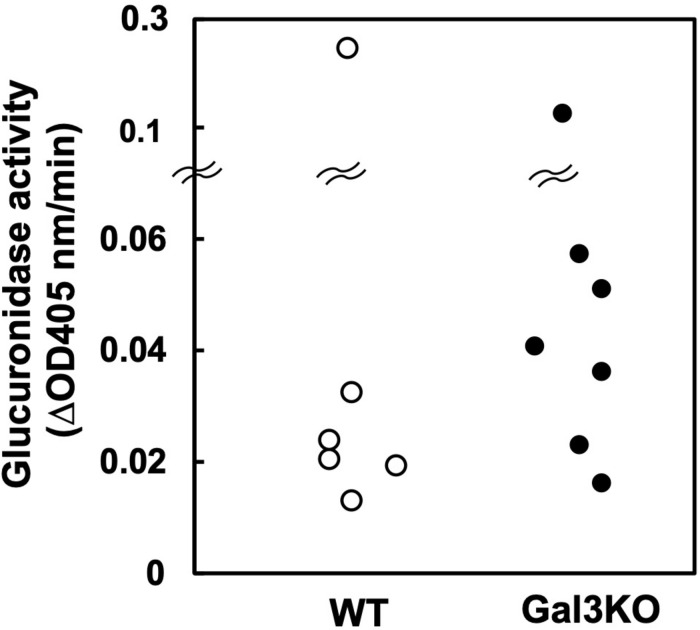
Glucuronidase activity of fecal bacteria. Fecal suspension from naïve WT (○) and Gal3KO mice (●) were prepared (*n* = 7). To determine β-glucuronidase activities, we incubated 20 μL of the fecal suspension with 180 μL of the reaction mixture containing 1 mM *p*-nitrophenyl-β-D-glucuronide, 50 mM HEPES-HCl (pH 7.4), and 37.2 mM 2-mercaptoethanol at 37°C for 30 min. The optical density (OD) was measured at 405 nm every 5 min (24). ΔOD: OD changes per 1 min.

Indomethacin is inactivated in the liver by glucuronidation and excreted in bile, and then, in the intestine, glucuronides-Indo are cleaved by bacterial β-glucuronidase, releasing free Indo. Subsequently, the enterocytes are exposed to relatively high concentrations of free Indo (17). Bacterial β-glucuronidase activities differ amongst bacterial species (24); several intestinal bacteria species in the families *Lachnospiraceae* and *Bacteroidaceae* have β-glucuronidase. Inhibiting the β-glucuronidase activity has been reported to protect mice against Indo-induced small intestinal ulcers (25). Thus, we compared the β-glucuronidase activities of fecal samples between WT and Gal3KO mice. We found no significant differences between the two mouse groups (Figure 4), suggesting that the β-glucuronidase levels are irrelevant to the suppression of Indo-induced ulcers in Gal3KO mice.

Non-steroidal anti-inflammatory drug-induced intestinal ulcers are known to occur by the mucosal invasion of intestinal bacteria (25, 26). Thus, we harvested small intestinal contents from WT and Gal3KO mice, isolated bacterial DNA, and conducted a 16S rRNA-based microbiome analysis. We compared the intestinal microbiomes between naïve WT and Gal3KO mice. At the family level, the intestinal microbiomes were mainly composed of the family *Lactobacillaceae* in both WT and Gal3KO mice (Supplementary Figure 1). Other than *Lactobacillaceae*, the families *S24-7* and *Bifidobacteriaceae* were highly abundant in WT mice; the *Clostridiales* order unknown family and the family *Streptococcaceae* were highly abundant in G3KO mice (Supplementary Figure 1).

Pathogenic or protective roles of individual bacteria in NSAID-induced small intestinal ulcers are not clear, although treatment with lactic acid-generating bacteria, including *Lactobacillus*, has been shown to change the intestinal microbiota, reducing intestinal ulcers (27, 28). To determine the roles of the intestinal microbiota, we first attempted to use co-housing of WT and Gal3KO mice as a means to transfer the microbiota, since co-housing has been shown to efficiently merge/exchange the fecal microbiota of the two mouse strains (29). Nevertheless, we found that there were significant differences in the small intestinal microbiota between WT and Gal3KO mice following co-housing. Thus, in our experimental system, fecal transplantation or coprophagy cannot be used for the equalization of the small intestinal microbiota.

**FIGURE 5:**
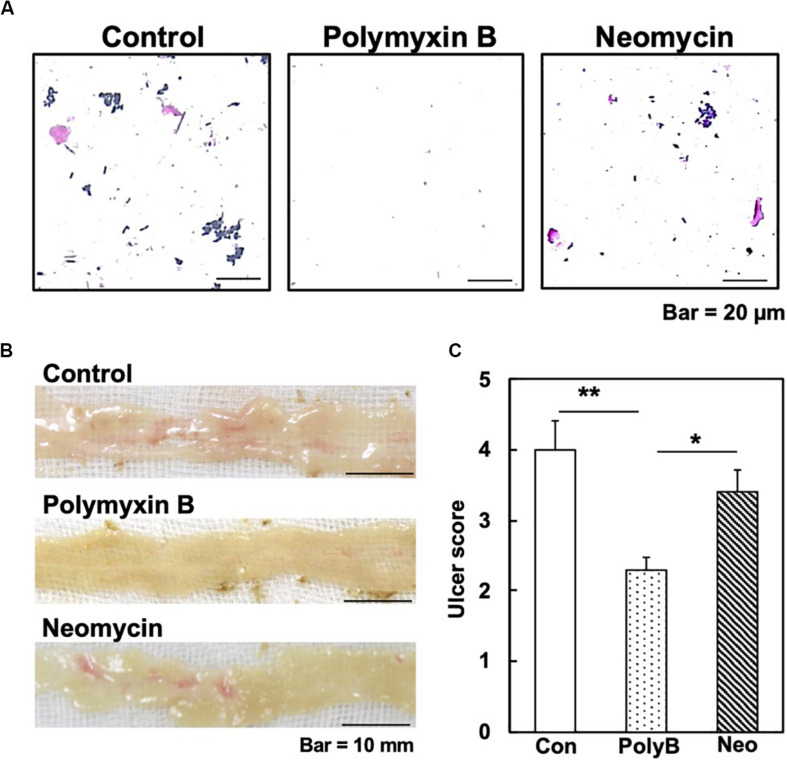
Small intestinal microbial reduction by antibiotics and Indo-induced intestinal ulcers in WT mice. We treated WT mice with unabsorbable antibiotics, polymyxin B (PolyB, 0.5 mg/mL, Pfizer Inc., New York, NY, United States) or neomycin (Neo, 1 mg/mL, Thermo Fisher Scientific, Waltham, MA, United States) in drinking water. **(A)** On day 6, we killed mice and harvested small intestinal contents. We prepared suspensions of the contents in water and conducted Gram staining (FUJIFILM Wako). **(B)** On day 6, Indo was administrated, and 18 h later, we harvested, rinsed the small intestine, and took macroscopic images (*n* = 5). Representative images of the jejunum. Bar = 10 mm. **(C)** PolyB-treated mice had lower ulcer scores than the control (Con) and Neo-treated mice. Ulcer severities were determined by the modified ulcer scores. **P < 0.01; *P < 0.05 by the ANOVA with *post hoc* Tukey test.

Thus, to determine the extent of which intestinal microbiota could affect Indo-induced ulcers, we used an alternative protocol, treating mice with either the bactericidal antibiotic polymyxin B (PolyB) or bacteriostatic antibiotic neomycin dissolved in drinking water for 6 days before induction of ulcers (30). We confirmed the effects of the two antibiotics on the intestinal bacteria by conducting Gram staining of small intestinal contents harvested from the WT mice treated with the antibiotic, compared with those from the untreated control mice. In the control mice without antibiotics treatment, the majority of the bacteria were Gram-positive bacilli. We observed substantial reductions of small intestinal bacteria in the PolyB-treated group, although the effect of neomycin was limited (Figure 5A). Then, we assessed the levels of ulceration in the mice treated with each antibiotic. We found that ulceration of the PolyB-treated group was less severe than the control group. On the other hand, the neomycin-treated group had a mild decrease in ulceration compared with the control group (Figures 5B,C). Thus, our results further support the role of the gut microbiota in NSAID-induced ulcers, reported by other research groups who demonstrated the suppression of NSAID-induced intestinal ulcers by treatment with antibiotics including ampicillin and aztreonam (24, 25).

In the above experiments, we found that the amount of bacterial DNA in the jejunum contents from PolyB-treated mice decreased in 60% of that from untreated control mice, suggesting that the reduction of bacteria could contribute to the decrease in ulceration of PolyB-treated mice. Since the changes in bacterial composition can also affect the NSAID-induced ulcers, using 16S rRNA sequencing of bacterial DNA, we compared the microbiomes in the jejunum contents between the PolyB-treated and control groups, using principal component analysis (Supplementary Figure 2) and alpha diversity indexes (Supplementary Figures 2, 3). Although there were no significant differences in alpha diversity of the microbiomes between the two groups (Supplementary Figure 3), PCA clearly separated the samples from the two groups into distinct populations (Supplementary Figure 2A), where there were significant differences in principal component (PC) 1 values between the two groups (*P* < 0.01, Supplementary Figure 2B). Factor loading for PC1 showed that a decrease in the family *Bacteroidaceae* (order *Bacteroidetes*, Gram-negative) and an increase in the family *Desulfovibrionaceae* (order *Proteobacteria*, Gram-negative) correlated to PC1 values.

These results were consistent with the previous findings that Gram-negative bacilli including some species of *Bacteroidetes* were susceptible to PolyB (31), but some species of *Desulfovibrionaceae* had low sensitivity to PolyB (32). Previously, the high abundance (33–35) and the colitogenic property (36) of *Bacteroidaceae* have been reported in animal models of colitis. *Bacteroidaceae* can also enhance the immune reaction by activating dendritic cells (35). On the other hand, *Desulfovibrionaceae* has been reported to decrease in the feces of the DSS-induced colitis model and seemed to have protective effects against the inflammation (37). *Desulfovibrionaceae* can also produce hydrogen sulfide (H_2_S), which plays roles in vasodilation and anti-inflammation in the gut (38). Thus, the changes in bacterial composition by PolyB treatment could be beneficial in the NSAID-induced ulcers.

### Role of Macrophages in NSAID-Induced Ulcers

**FIGURE 6:**
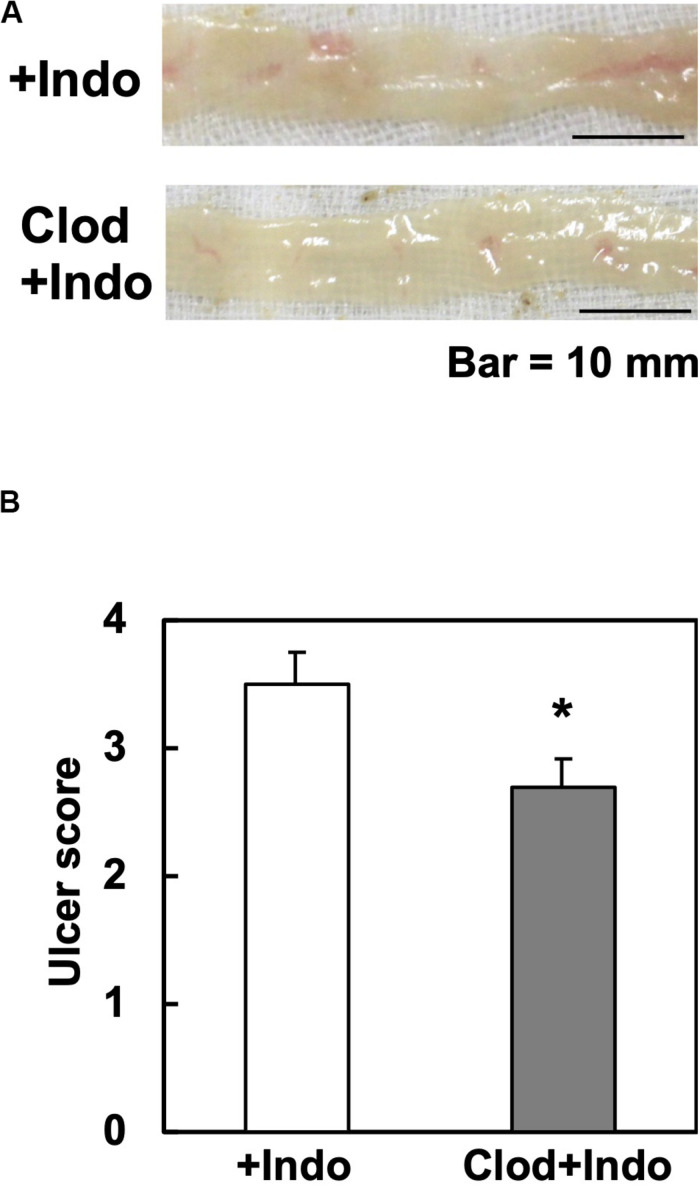
Clodronate injection and Indo-induced intestinal ulcers in WT mice. We injected clodronate (Clod) intraperitoneally into WT mice. The next day, we administered Indo and killed mice 18 h later. We harvested and rinsed the small intestines, and took macroscopic images (*n* = 5). **(A)** Representative images of the jejunum from Indo-administered mice without Clod treatment (+Indo) showed more severe ulcers than mice treated with Clod (Clod+Indo). Bar = 10 mm. **(B)** Ulcer severities were determined by the modified ulcer scores. *P < 0.05 by the Mann–Whitney *U* test.

Since the Gal3 expression in macrophages has been reported to potentiate immune responses, we tested whether macrophage depletion could attenuate Indo-induced ulcers. Since clodronate, particularly its liposome-encapsulated form, has been used to deplete macrophages, we injected liposome-encapsulated clodronate, MacrokillerV300 (Cosmo Bio Co., Ltd., Tokyo, Japan), into mice. As observed by others, however, Macrokiller treatment alone caused severe body weight loss and diarrhea in the mice; we were unable to conduct an additional injection of Indo. Since the injection of clodronate itself (30 mg/kg, peritoneally, Tokyo Chemical Industry Co., Ltd., Tokyo, Japan) did not result in body weight changes or diarrhea, we treated mice with clodronate, instead of Macrokiller, 1 day before Indo administration. We found that the clodronate injection reduced the ulcer levels, mildly but significantly (Figure 6).

Since the number of small intestinal macrophages quantified by flow cytometry did not differ between the clodronate-injected versus control mice (data not shown), the mechanism by which clodronate suppressed the intestinal ulceration was unlikely due to the depletion, but suppression of macrophages functionally. Inhibition of macrophage-lineage cells by clodronate, a first-generation bisphosphonate, has been demonstrated. Experimentally, clodronate inhibits expression of tumor necrosis factor (TNF)-α and interleukin (IL)-6 in macrophages (39), suppressing inflammation. Clinically, clodronate has been prescribed for osteoporosis (32, 33); the pharmacological mechanism of clodronate is to inhibit the osteoclast activity with no macrophage-killing activity. Although clodronate has been shown not to suppress neutrophils directly (39), clodronate may affect neutrophils that infiltrate into Indo-induced ulcers indirectly; in theory, extracellular Gal3 released from macrophages could promote neutrophil functions (9, 10).

## Discussion

In this study, we found that Indo-induced ulcers were less severe in Gal3KO mice than in WT mice. We propose two mechanisms by which Gal3 could exacerbate Indo-induced intestinal ulcers; (1) the Gal3 expression in the small intestinal epithelia alters the bacterial population in the jejunum, enhancing mucosal bacterial invasion; and (2) the Gal3 expression in activated macrophages enhances inflammation. Consistently, we found that suppression of Indo-induced intestinal ulcers in WT mice seemed to be associated with both (1) gut microbial reduction and alteration by antibiotics treatment and (2) macrophage suppression by clodronate treatment. However, the levels of the ulcer suppression by the two treatments were not comparable to that of Gal3 KO mice (average ulcer scores: control WT group, 3 to 4; antibiotics WT group, 2.3; clodronate WT group, 2.7; and Gal3KO group, 0.4) (Figures 2, 5, 6). Thus, our two proposed mechanisms may be closely related, and not mutually exclusive; for example, the enhanced bacterial invasion could activate macrophages. Although probiotics including *Lactobacillus* as well as macrophage inhibitors including clodronate can be useful to suppress the ulcers to some extent, Gal3 inhibition in both intestinal epithelia and macrophages is ideal for more efficient suppression of NSAID-induced small intestinal ulcers.

Galectin-3 affects numerous biological processes and has been suggested to play either beneficial or detrimental roles in several chronic diseases: amelioration of microbial infection (9, 33) and kidney function (40); exacerbation of atherosclerosis (35, 36), lung and renal fibrosis (14, 37), non-alcoholic steatohepatitis (NASH) (16, 38), and cancers (17, 39). Since most of the conditions listed above are associated with chronic diseases, it will be possible to minimize the adverse effects by prophylactic Gal3 inhibition for NSAID-induced small intestinal ulcers, if Gal3 inhibitors are used only for the short term. Indeed, galectin inhibitors, which inhibit Gal3 predominantly and Gal1 partially, have already been shown to be safe and applicable in several disease conditions, clinically and experimentally. Recently, TD139 also known as GB0139 has attracted attention as a highly specific Gal3/Gal1 inhibitor: the affinity of TD139 for Gal3 with *K*_*d*_ = 14 nM and for Gal1 with *K*_*d*_ = 10 nM, but low for galectins 2, 4, 7, 8, or 9 (41). Currently, TD139 is used in a clinical trial for idiopathic pulmonary fibrosis (IPF). After successful results of Phase I/II trials which assessed the safety and tolerability in healthy volunteers and patients with IPF, Phase IIb clinical trial of TD139 treatment is now enrolling up to 450 eligible IPF patients at sites across the United States, Canada, Europe, United Kingdom, and Israel.

## Conclusion

We demonstrated that inhibition of Gal3 could be a therapeutic strategy in NSAID-induced intestinal ulcers. Although targeting this molecule may cause several unexpected outcomes because of its variety of functions, Gal3 inhibitors, particularly for its short-term use, have already been shown to be safe, clinically and experimentally. The future invention of the Gal3 inhibitor that can target only a specific organ or cell type should be safe and effective to treat a variety of disease conditions; an intestine-specific Gal3 inhibitor could reduce NSAID-induced intestinal ulcers without adverse effects.

## Materials and Methods

### Mice

We purchased CD1 mice from Charles River Laboratories Japan (Yokohama, Japan). The generation of Gal3KO mice was described previously (12). The animal experiments were approved by the Institutional Animal Care and Use Committee of Kindai University (Osaka, Japan) and performed in accordance with the institutional guidelines.

### Immunological Staining, PAS Staining, and HE Staining

We made 4-μm-thick tissue sections. Immunohistochemistry (IHC) was performed by the standard procedure using Histofine SAB-PO kit (Nichirei Biosciences; Tokyo, Japan) and 3,3’-diaminobenzidine (DAB). Polyclonal antibodies used for IHC were as follows: anti-Gal3 (BioLegend, San Diego, CA, United States), anti-F4/80 (AbD Serotec, Kidlington, United Kingdom), and anti-Ly6/G (BD Biosciences, San Jose, CA, United States). PAS and hematoxylin and eosin (H&E) staining were performed by the standard procedures.

### Induction and Assessment of Indo-Induced Small Intestinal Ulcers

Indo (FUJIFILM Wako, Osaka, Japan) was dissolved in a 0.5% NaHCO_3_ solution and administrated to non-fasted mice by oral gavage. Control mice were administered a 0.5% NaHCO_3_ solution alone. We determined the Indo dosage (5 mg/kg body weight) by “Human equivalent dose calculation” (42). At 18 h after the injection, we collected feces, took blood from the right ventricle, and perfused the mice with phosphate-buffered saline (PBS) from the left ventricle. We harvested and rinsed the small intestines with PBS, opened along the anti-mesenteric side and took macroscopic images with a scale by a digital camera (Canon, Tokyo, Japan). Then, tissue pieces were fixed in formalin and embedded in paraffin for histological examinations.

The ulcer severity was evaluated using the ulcer score (23) with modification. The macroscopic ulceration areas were captured and quantified by an ImageJ software (NIH, Bethesda, MD, United States) and summed per jejunum. The entire jejunum areas were similar in all mice examined and were around 1,200 mm^2^. We used the modified ulcer score as follows: 0 = no lesion, 1 = less than 5 mm^2^, 2 = 5∼20 mm^2^, 3 = 21∼40 mm^2^, 4 = 41∼70 mm^2^, and 5 = more than 71 mm^2^.

Fecal occult blood level was determined as described previously (43). Feces were suspended in distilled water and centrifuged at 12,000 × *g*. The supernatant was mixed with a luminol reagent (FUJIFILM Wako), and then chemiluminescence was measured by a luminometer (Wallac ARVO SX 1420 multilabel counter, PerkinElmer, Waltham, MA, United States).

### β-Glucuronidase Activities of Feces

To determine β-glucuronidase activities, we mixed 20 μL of the fecal suspension with 180 μL of the reaction mixture containing 1 mM *p*-nitrophenyl-β-D-glucuronide (FUJIFILM Wako), 50 mM HEPES-HCl (pH 7.4), and 37.2 mM 2-mercaptoethanol (24). We incubated the mixture at 37°C and measured its optical density (OD) at 405 nm, every 5 min between 10- and 30-min incubation times. We calculated the average OD change per min.

### Antibiotics Treatment and Gram-Staining

We treated WT mice with an unabsorbable antibiotic, polymyxin B (PolyB, 0.5 mg/mL, Pfizer Inc., New York, NY, United States) or neomycin (Neo, 1 mg/mL, Thermo Fisher Scientific, Waltham, MA, United States) in drinking water (*n* = 9 per group). On day 6, we killed four mice in each treatment group and harvested small intestinal contents. We prepared suspensions of the contents in water and conducted Gram staining (FUJIFILM Wako). Using the remaining five mice in each treatment group, we administered Indo and assessed the ulcer levels, using the ulcer score.

### Effect of Clodronate Injection on Indo-Induces Ulcers

One day before Indo administration, we injected clodronate (30 mg/kg body weight, FUJIFILM Wako) intraperitoneally into WT mice. Then, we administered Indo and assessed the ulcer levels, using the ulcer score.

## Data Availability Statement

The raw data supporting the conclusions of this article will be made available by the authors, without undue reservation.

## Ethics Statement

The animal study was reviewed and approved by the Institutional Animal Care and Use Committee of Kindai University.

## Author Contributions

A-MP designed and conducted the experiments. IT supervised the project. A-MP and IT wrote the manuscript. SK performed the microbiome analysis. FS, SO, MF, DKH, and F-TL conceived and conducted some experiments. All authors read and approved the final manuscript.

## Conflict of Interest

The authors declare that the research was conducted in the absence of any commercial or financial relationships that could be construed as a potential conflict of interest.
