# Supplementary material for: Galectin-3 as a Therapeutic Target for NSAID-Induced Intestinal Ulcers
Source: Front Immunol. 2020 Sep 23;11:550366. doi: 10.3389/fimmu.2020.550366 (PMC7539695; doi:10.3389/fimmu.2020.550366)
Supplement: Supplementary file 1 [file Data_Sheet_1.PDF]

Supplementary Materials and Methods

Microbiota analysis: In Suppl Fig 1, we harvested small intestinal contents from naïve wild-type (WT) and Gal3 knockout (Gal3KO) mice (n=2 each). In Suppl Figs. 2 and 3, we harvested small intestinal contents from WT mice with (n=3) or without polymyxin B treatment (n=4). Bacterial DNA was isolated and 16S rRNA-based microbiota analysis was performed by Takara Bio Inc (Shiga, Japan) or MR DNA (Shallowater, TX, USA).

Principal component analysis (PCA): We conducted PCA to compare the overall microbiota among the samples, using an R program 'prcomp', as we described previously (1). Factor loading for principal component (PC) was used to rank individual bacteria whose relative abundance was correlated with PC1 values. We drew a graph of PCA using R packages 'dplyr' and 'ggplot2'.

Alpha diversity: We compared alpha diversity of microbiota between the control and the polymyxin B-treated groups. Using R, we compared the number of families, evenness and combination of them by Chao 1 richness index, Pielou's evenness index, and Shannon-Wiener diversity index, respectively (2).

Supplemental References:

1. Omura S, Sato F, Park A-M, Fujita M, Khadka S, Nakamura Y, Katsuki A, Nishio K, Gavins FNE, and Tsunoda I. (2020). Bioinformatics analysis of gut microbiota and CNS transcriptome in virus-induced acute myelitis and chronic inflammatory demyelination; Potential association of distinct bacteria with CNS IgA upregulation. *Front Immunol* 2020 Jul 7;11:1138. doi: 10.3389/fimmu.2020.01138. eCollection 2020.
2. Xia Y, Sun J, Chen D-G (2018). Statistical Analysis of Microbiome Data with R. London, UK: Springer Nature.
