# Supplementary material for: Galectin-3 as a Therapeutic Target for NSAID-Induced Intestinal Ulcers
Source: Front Immunol. 2020 Sep 23;11:550366. doi: 10.3389/fimmu.2020.550366 (PMC7539695; doi:10.3389/fimmu.2020.550366)
Supplement: Supplementary file 2 [file Data_Sheet_2.pdf]

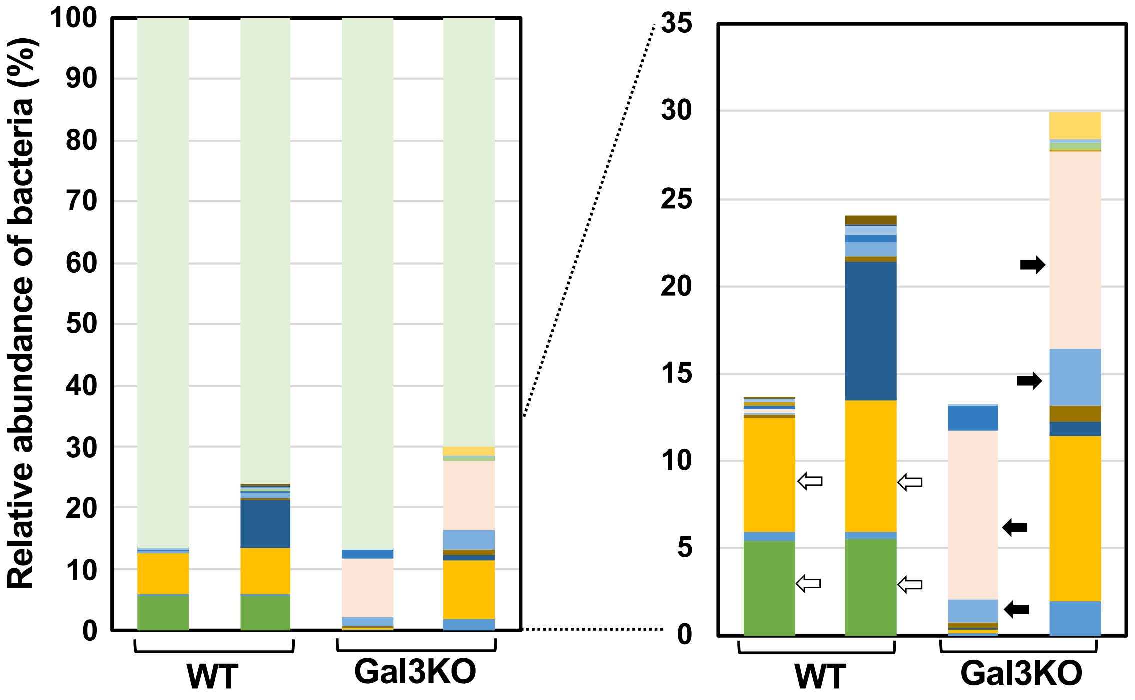

Phylum	Class	Order	Family	Gram
Firmicutes	Bacilli	Lactobacillales	Lactobacillaceae	+
Verrucomicrobia	Verrucomicrobiae	Verrucomicrobiales	Verrucomicrobiaceae	−
Proteobacteria	Gammaproteobacteria	Pasteurellales	Pasteurellaceae	−
Proteobacteria	Gammaproteobacteria	Enterobacteriales	Enterobacteriaceae	−
Proteobacteria	Deltaproteobacteria	Desulfovibrionales	Desulfovibrionaceae	−
Proteobacteria	Betaproteobacteria	Burkholderiales	Alcaligenaceae	−
Firmicutes	Erysipelotrichi	Erysipelotrichales	Erysipelotrichaceae	+
Firmicutes	Clostridia	Clostridiales	Lachnospiraceae	+
Firmicutes	Clostridia	Clostridiales	Clostridiaceae	+
Firmicutes	Clostridia	Clostridiales	−	+
Firmicutes	Clostridia	Clostridiales	Other	+
Firmicutes	Bacilli	Lactobacillales	Streptococcaceae	+
Firmicutes	Bacilli	Lactobacillales	Leuconostocaceae	+
Firmicutes	Bacilli	Gemellales	Gemellaceae	+
Firmicutes	Bacilli	Bacillales	Staphylococcaceae	+
Cyanobacteria	Chloroplast	Streptophyta	−	−
Bacteroidetes	Bacteroidia	Bacteroidales	S24-7	−
Actinobacteria	Coriobacteriia	Coriobacteriales	Coriobacteriaceae	+
Actinobacteria	Actinobacteria	Bifidobacteriales	Bifidobacteriaceae	+

⇦ (light green arrow) indicates the family *Lactobacillaceae.*
⇦ (white) and ⬅ (black) arrows indicate bacteria of high relative abundance in WT mice and Gal3KO mice, respectively. Gram, Gram staining.

Supplemental Figure 1. Small intestinal microbiota in WT and Gal3KO mice
We harvested small intestinal contents from naïve wild type (WT) and Gal3 knockout (Gal3KO) mice (n=2 each). Bacterial DNA isolation and 16S rRNA-based microbiota analysis was performed. (Left) Inter individual variation of total microbiome of each mouse at the family level, which was mainly composed of the family *Lactobacillaceae* (light green). (Right) Other than the family *Lactobacillaceae*, the families *S24-7* (orange) and *Bifidobacteriaceae* (green) were high in WT mice; the *Clostridiales* order unknown family (light pink) and the family *Streptococcaceae* (light blue) were high in Gal3KO mice.

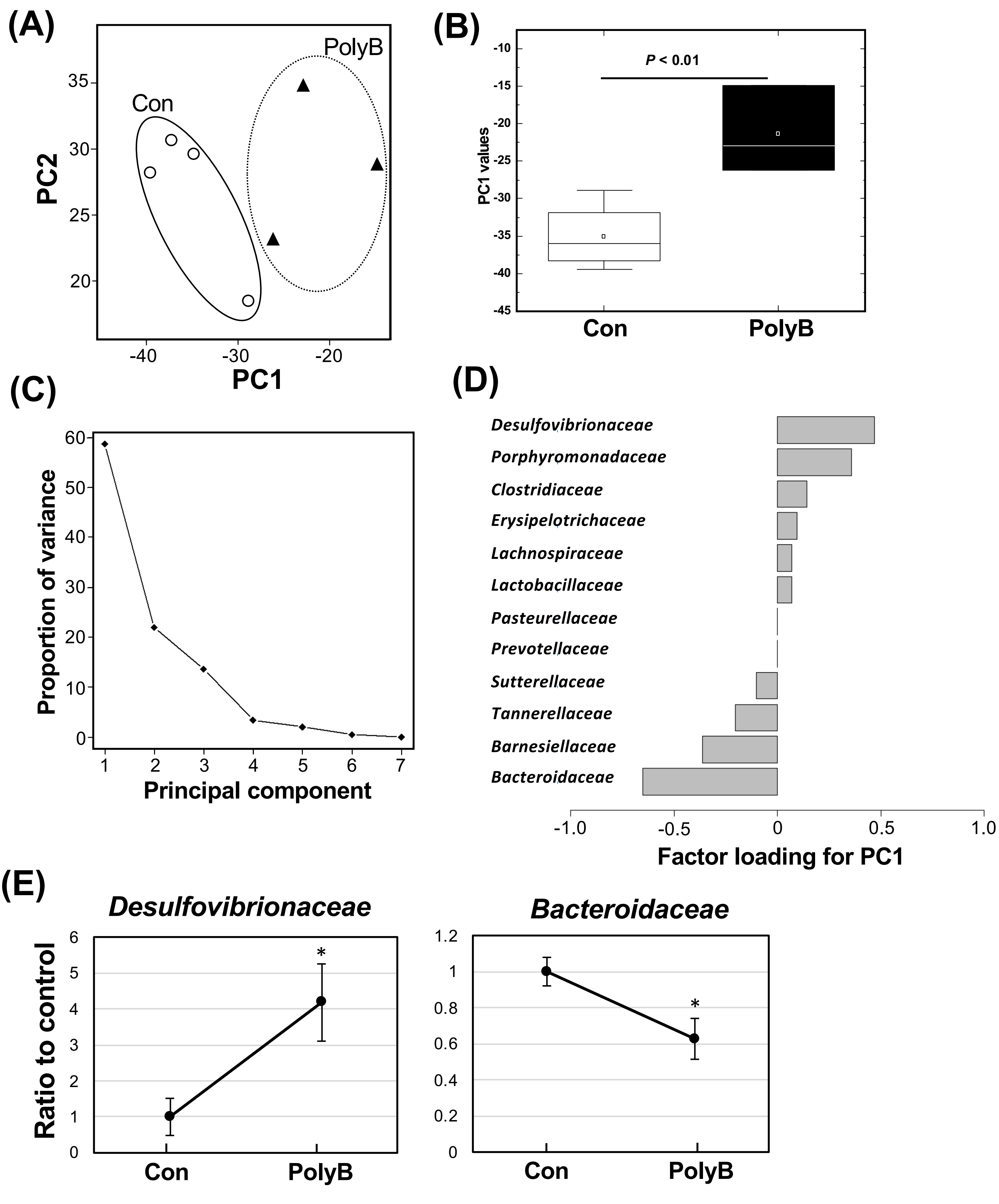

Supplemental Figure 2. Small intestinal microbiota in control and polymyxin B treated WT mice. **(A)** Principal component analysis (PCA) of microbiome data from control (Con, ○) and polymyxin B (PolyB)-treated mice (▲). **(B)** Two groups were separated by principal component (PC)1 values ($P < 0.01$). We conducted PCA, using microbiome data at the family level. **(C)** Proportion of variance for PC1 and PC2 accounts for 58% and 22%, respectively. **(D)** Factor loading for PC1 showed that relative abundance of families *Desulfovibrionaceae* and *Bacteroidaceae* were positively and negatively correlated with PC1 values, respectively. **(E)** Relative changes of *Desulfovibrionaceae* and *Bacteroidaceae* by PolyB treatment. *$P < 0.05$ Con versus PolyB treatment by the Student *t*-test.

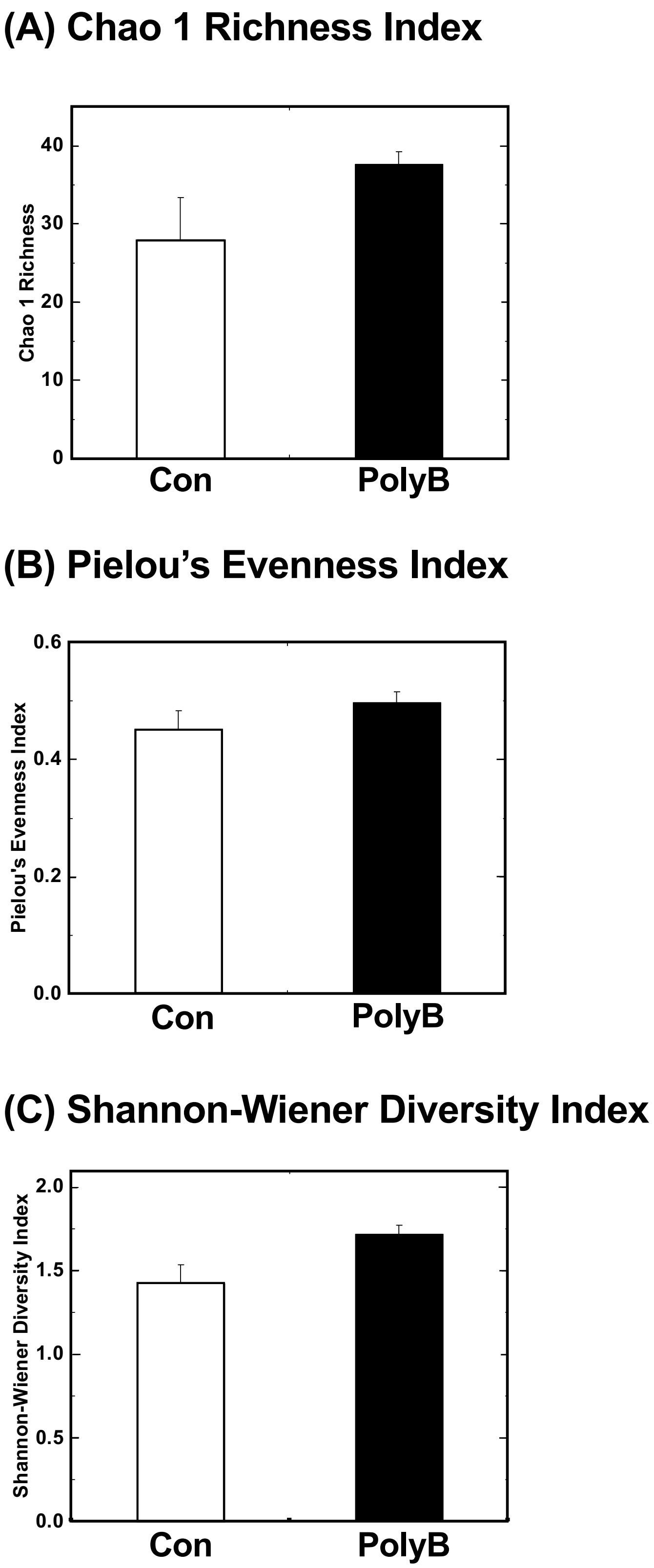

Supplemental Figure 3. Analyses of bacterial alpha diversities of microbiome between control and polymyxin B-treated groups. Using R, we compared the number of families, evenness and combination of them between the control (con) and the polymyxin B (PolyB)-treated groups by Chao 1 richness index **(A)**, Pielou's evenness index **(B)**, and Shannon-Wiener diversity index **(C)**, respectively (1). We didn't find significant differences between the groups.

(1) Xia Y, Sun J, Chen D-G (2018). Statistical Analysis of Microbiome Data with R. London, UK: Springer Nature.
